# Jejunal Intussusception in Adolescent Crohn's Disease: An Extremely Rare Complication

**DOI:** 10.1155/2020/8880100

**Published:** 2020-09-12

**Authors:** Narendra Pandit, Sujan Gautam, Tek Narayan Yadav, Lawa Kumar Mandal, Kunal Bikram Deo

**Affiliations:** Division of Surgical Gastroenterology, Department of Surgery, B P Koirala Institute of Health Sciences (BPKIHS), Dharan, Nepal

## Abstract

Proximal small bowel intussusception occurring in an adolescent Crohn's disease patient is an extremely rare entity. It is usually primary without a lead point and quite often a transient phenomenon. We report such transient and intermittent jejunal intussusception in a 16-year-old male, developing immediately in a postoperative period after a stoma reversal for jejunal stricture perforation peritonitis.

## 1. Introduction

Crohn's disease is a chronic granulomatous inflammatory disease that involves any part of the gut from the mouth to the anus. Terminal ileum is the most commonly (70%) involved location of the bowel [1]. It has been associated with varied presentation and complication ranging from abdominal pain, fistulae, abscess, perforation, stricture, failure to thrive, and bleeding [2, 3]. Intestinal obstruction secondary to jejunal intussusception by a segment of active Crohn's disease in adolescents has been rarely reported in the literature [4, 5]. Here, we describe a rare case of transient jejunojejunal intussusception in a postoperative setting following stoma closure.

## 2. Case Report

A 16-year-old male underwent exploratory laparotomy, jejunal and ileal resection (50 cm) for jejunal stricture-perforation peritonitis with double-barrel jejunostomy at an outside hospital (by general surgeon) 3 months back. Approximately 30 cm of distal jejunal and 20 cm of the proximal ileal segment, which was grossly diseased, were resected (without any anastomosis). Intraoperatively, there were multiple passable strictures in the jejunum and the ileum. However, the ileocecal junction and the colon were normal (Montreal classification- A1L1B3). Histopathology report of the resected specimen confirmed Crohn's disease.

**Figure 1:**
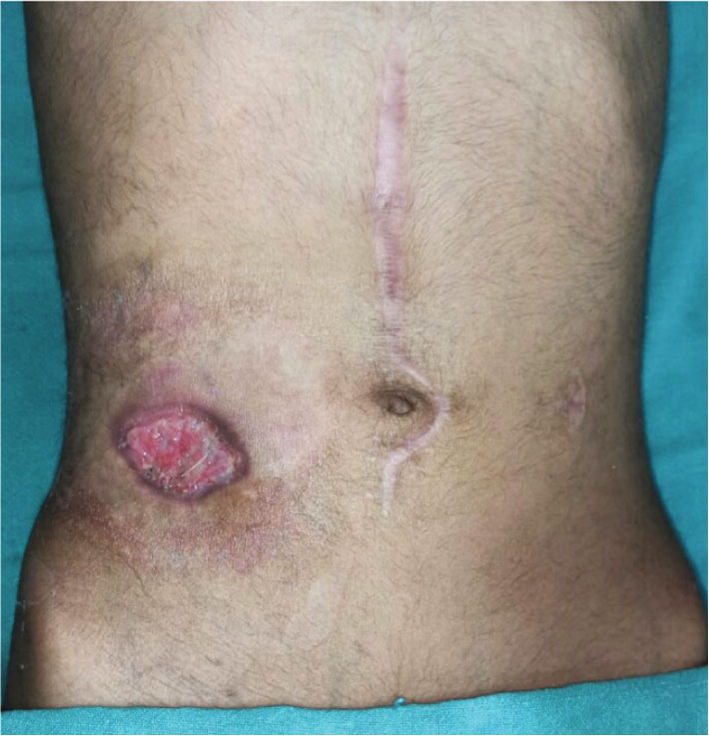
Image showing nondistended abdomen with a previous midline laparotomy scar and a healing wound of stoma closure site.

**Figure 2:**
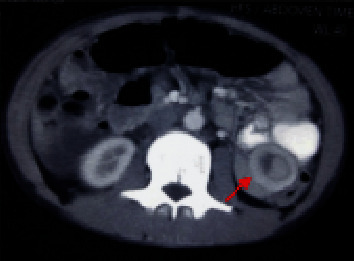
Computed tomography scan demonstrates jejunojejunal intussusception with a target sign (arrow) and minimal dilated proximal jejunal loop.

This time, he was referred to our center (gastrointestinal surgery unit) for early stoma closure due to the high stoma output (4 to 5 liters per day) and further management. The patient underwent double-barrel jejunostomy reversal from the stoma site (Figure 1) after optimization of the patient. Following stoma closure, he had been taking a liquid diet and doing well for the last 12 days during the hospital stay. On the 13^th^ day, the patient complained of pain abdomen. The pain was colicky, intermittent, mild in severity, and located in the left hypochondrium. He denied any history of vomiting, fever, or obstipation. On examination, the patient was comfortable, with tachycardia (104/min), but normal blood pressure. The abdomen was mildly distended without any tenderness or guarding. Blood investigation revealed leukocytosis (22,000 cells/mm^3^). Abdominal X-ray showed ground glass appearance with paucity of an air-fluid level. Abdominal ultrasonography showed no free fluid in the abdomen. Contrast computed tomography of the abdomen was done, which revealed jejunojejunal intussusception (Figure 2). There were no features of delayed anastomotic leak or vascular compromise of the jejunum. The patient was managed conservatively with nil per oral, intravenous fluids, parenteral nutrition, and antibiotics. The patient improved with it over the next two days. The abdomen distention settled, leukocytosis came to normal, and an intussusception resolved as confirmed by an abdominal ultrasound. The patient was allowed orally, tolerated and passed stool, and discharged on day 20. On follow-up, the patient is doing well, initiated on anti-TNF therapy, without any symptom recurrence.

## 3. Discussion

Intussusception in Crohn's disease though described is a rare event [4]. However, it can occur on the small bowel because of the dysrhythmic contraction secondary to the ongoing inflammatory process of a thickened, inflamed segment of bowel wall. The inflammatory edema, spasm, enlarged mesenteric lymph nodes, small bowel polyp lead to impaired contraction of the bowel, and allowed unbalanced peristaltic forces to rotate the bowel wall inwards and initiate the invagination. Hence, the majority does not have a demonstrable etiology as the lead point [4, 6].

The most common site for the intussusception is the ileocecal region, and the jejunojejunal or ileoileal is the rare site. Most importantly, it can be a transient phenomenon in the absence of vascular compromise of the bowel as seen in the present case [7]. Furthermore, postoperatively after stoma reversal, it can occur due to initiation of proper peristaltic activity of the defunctionalized bowel. Thus, the diagnosis is often difficult and delayed because clinical symptoms are not specific and the diagnosis is performed mainly by imaging studies [8].

As Crohn's disease cannot be cured and is a recurrent disease requiring, multiple surgeries, bowel resection with risks of short bowel syndrome, mere presence of small bowel intussusception does not require laparotomy [9]. As the event can be transient, spontaneous resolution can occur with conservative management. Even though operative intervention is required, manual reduction is preferable over resection unless the bowel is strangulated to avoid stoma, anastomotic leak, and short bowel syndrome [1, 10]. Hence, there should be high threshold for operative intervention in these patients with jejunal intussusception.

To conclude, jejunojejunal intussusception in a postoperative period following stoma reversal for active Crohn's disease is exceedingly rare and can be a transient phenomenon. High threshold for operative intervention in this group of patients is required to avoid a series of complications related to the disease.

## Data Availability

The data used to support the findings of this study are included within the article.
